# Cognitive Benefits of Activity Engagement among 12,093 Adults Aged over 65 Years

**DOI:** 10.3390/brainsci10120967

**Published:** 2020-12-10

**Authors:** Jieting Zhang, Liye Zou, Can Jiao, Minqiang Zhang, Lina Wang, Wook Song, Qian Yu, Igor Grabovac, Yanjie Zhang, Peter Willeit, Lin Yang

**Affiliations:** 1 Institute of Mental Health, School of Psychology, Shenzhen University, Shenzhen 518060, China; jennyzhang@szu.edu.cn (J.Z.); liyezou123@gmail.com (L.Z.); wanglina2018@email.szu.edu.cn (L.W.); 2 Exercise and Mental Health Laboratory, School of Psychology, Shenzhen University, Shenzhen 518060, China; yuqianmiss@163.com; 3 Center for Studies of Psychological Application, South China Normal University, Guangzhou 510631, China; zhangminqiang@m.scnu.edu.cn; 4 School of Psychology, South China Normal University, Guangzhou 510631, China; 5 Guangdong Psychological Association, Guangzhou 510631, China; 6 Health & Exercise Science Laboratory, Institute of Sports Science, Department of Kinesiology, Seoul National University, Seoul 08826, Korea; songw3@snu.ac.kr (W.S.); elite_zhangyj@163.com (Y.Z.); 7 Institute on Aging, Seoul National University, Seoul 08826, Korea; 8 Department of Social and Preventive Medicine, Center for Public Health, Medical University of Vienna, Kinderspitalgasse 15/1, 1090 Vienna, Austria; igor.grabovac@meduniwien.ac.at; 9 Physical Education Unit, School of Humanities and Social Science, the Chinese University of Hong Kong-Shenzhen, Shenzhen 518172, China; 10 Department of Neurology, Medical University of Innsbruck, 6020 Innsbruck, Austria; peter.willeit@i-med.ac.at; 11 Department of Public Health and Primary Care, University of Cambridge, Cambridge CB2 1TN, UK; 12 Department of Cancer Epidemiology and Prevention Research, Cancer Care Alberta, Alberta Health Services, Calgary, AB T2S 3C3, Canada; lin.yang@albertahealthservices.ca; 13 Departments of Oncology and Community Health Sciences, Cumming School of Medicine, University of Calgary, Calgary, AB T2N 1N4, Canada

**Keywords:** cognition, activity, older adults, aging, lifestyle

## Abstract

Objective: The present study includes two aims: (1) to understand patterns of activity engagement among older Chinese adults; (2) to further investigate associations between activity engagement and cognitive abilities in this population. Methods: Latent class analysis was applied to answer the aforementioned research questions across different age ranges while controlling for confounding variables (age, health, socioeconomic status (SES), and living alone). Specifically, five latent classes (non-active, working-active, comprehensive-active, physical-active, and less-active) were identified. Furthermore, associations between the classes of activity engagement and cognition were examined separately in three age groups: less than 80 years (young-old group), 80–99.5 years (old-old group) and more than 100 years (oldest-old group) of age. Results: Compared with Non-active older individuals, the other classes with a higher probability of engagement in various activities generally showed higher cognitive abilities (including general cognition, orientation, calculation, recall, and language), but not all patterns of active engagement in daily life were positively associated with better cognitive status across different age ranges. In particular, differences in the individuals’ cognitive abilities across the four active latent classes were especially obvious in the old-old group as follows: the Comprehensive-active class had higher general cognitive and recall abilities than the other three active classes and higher calculation and language abilities than the Working-active class. In addition, significant sex differences were observed in activity patterns, cognition, and their associations in the young-old and old-old groups. Culture-specific programs should be customized to subgroups of different ages and genders by providing different training or activity modules based on their related dimensions of cognitive decline.

## 1. Introduction

Activity engagement, which is defined as how ageing individuals spend time engaging in daily activities, has been shown to be associated with cognitive function [1,2]. Older adults usually participate in more than one daily activity, and different types of activities may have differential effects on one or more aspects of cognition [3,4,5]. For instance, mental activities, such as reading, may protect against dementia and cognitive decline [6,7,8], and other individual activities, such as gardening and walking, may reduce the risk of dementia and Alzheimer’s disease [9]. Social activities (e.g., visual contact with relatives and community social integration) were positively related to happiness, physical function, and mortality, and engagement with friends was especially protective against cognitive decline in women, but not men [6,10].

Less is known about the pattern of engagement in these activities among older Chinese adults, and interactions between these patterns and cognitive ability have often been under-investigated in traditional regression models to avoid cumbersome parameters. Latent class analysis (LCA) [11] is a technique designed to identify subgroups of individuals with different behavioral patterns based on related categorical indicators. In addition to the quantitative amount of activity engagement, LCA provides additional information regarding the pattern of activity engagement in each latent class. Namely, people engaging in the same number of activities may participate in various types of activities (e.g., being active in exercising and doing housework versus being active in reading and engaging in social activities). Moreover, subsequent LCA simplifies higher-order interactions among indicators with relatively few subgroups and thus avoids high error rates and reduced statistical power when the relations between various activities and cognitive ability are investigated [12].

Despite increasing LCA studies of activity engagement for the elderly [13,14,15], only two studies have examined its association with cognitive function among older adults in the U.S. [16,17]. These studies provided evidence that older people participate in multiple activities and that the patterns of engagement are associated with different levels of cognition. For instance, subgroups with low activity were associated with cognitive impairment [16]. The subgroups with high engagement in all activities or in working showed better cognition outcomes compared with the subgroup engaged in passive leisure [17]. However, the previous literature only investigated the association of activity patterns with the total cognition score, rather than specific dimensions of cognitive ability. Additionally, further attention should be paid to older Chinese adults, as population aging has been accelerating in China [18] and cognitive decline in this population would be more serious due to the disadvantageous education they received [19]. Moreover, as compared to males, the risk of cognitive impairment is higher among females due to their lower early-life socioeconomic status (SES) in China [19], greater Aβ deposition [20], or/and their different activity patterns [6,10] Functional status, affective status, cognitive status, and productive involvement also vary across different stages of aging [21,22].

Considering the high heterogeneity in such a large population of older Chinese adults, differences in the activity–cognition link should be considered by gender and age range (i.e., young-old, old-old, and oldest-old) [23]. Hence, by using latent class modelling, the current study aimed to identify subgroups of older Chinese adults characterized by particular patterns of activity engagement and further estimate the association between these patterns and cognitive ability across different age ranges after controlling for related confounders (i.e., health, SES, and living alone). Moreover, we investigated the sex differences in activity pattern, cognition, and their association, which may have practical implication for the prevention of cognitive impairment. Results in this study would allow health professionals and policy makers to customize age-appropriate and gender-specific programs for cognitive benefits.

## 2. Method

### 2.1. Participants

The data used in this study were derived from the Chinese Longitudinal Healthy Longevity Survey (CLHLS) in 2008 [24,25]. The CLHLS was conducted on a randomly selected sample from counties and cities within [22] Chinese provinces. The survey area covered 85% of the Chinese population. Chinese adults aged 65 years and older were selected to participate in this study (*n* =12,093, *M*_age_ = 84.60 years (*SD* = 10.96, range 65–116), 46.7% male, 93.0% Han Chinese). Participants with dementia were excluded based on the clinical thresholds of the Mini-Mental State Examination (MMSE) for the elderly Chinese population (19.5 for males and 18.5 for females) [26]. Notably, the cut-off scores are lower than those in other countries to ensure better measurement validity [26] because of the high proportion of older individuals with limited education or illiteracy in this dataset [27]. All the variables were measured and collected in the same wave.

### 2.2. Outcome Measures

Activity engagement: Ten items were used to investigate the activities that older adults perform daily: reading newspapers/books, watching TV/listening to the radio, playing cards/mah-jong, participating in social activities, travelling, exercising, doing housework, raising livestock, farming and doing other farming work, and gardening. All the activities were assessed with the same question, namely “Do you do (activity) currently?”, and the responses were binary (yes = 1, no = 0). For example, the participants were asked, “Do you do housework currently?”

Cognitive ability: The cognitive abilities among the elderly participants were measured using the Chinese version of the MMSE [27], which was translated from the international standard of the MMSE questionnaire [28,29] and has been carefully tested in pilot survey interviews to localize all items. In this study, the MMSE tests four aspects of cognitive functioning: orientation, calculation, recall, and language [30], and item average scores for each of the four aspects were calculated. Responses of ‘‘unable to answer’’ were treated as missing data. The alpha coefficient of the MMSE for the current data was 0.91. Following the method of scoring in a previous study [27], we calculated the sum scores across all items [31,32] and for each sub-test (α = 0.74~0.89) to reflect global cognition.

Demographic variables: Data on sex (male = 1, female = 0), age, living alone (yes = 1, no = 0), SES, and health status were collected. Based on the indicators popularly used in previous studies [19,27], SES was assessed by years of education, urban residence (yes = 1, no = 0), and occupation (professional/administration = 1, otherwise = 0); and health status was determined by subjective health. Disability related to activities of daily living (ADL) was indexed by the number of ADLs from six items that a respondent could not perform independently: bathing, dressing, eating, indoor transferring, toileting, and continence (α = 0.89). Subjective health was assessed by asking the participants to rate their health status on a 5-point Likert scale (1 = “very bad”, 5 = “very good”).

### 2.3. Statistical Analysis

The latent class model was built by Mplus 7.4 (Muthén & Muthén, Los Angeles, CA, USA) based on the ten binary items of activity engagement to identify activity sub-types for older Chinese adults. Information criteria, entropy, parsimony, and interpretation of the classification were considered comprehensively to determine the best model. Specifically, lower akaike information criterion(AIC), bayesian information criterion(BIC) and sample-size adjusted BIC (nBIC) values indicate a better model fit, and higher entropy indicates a lower classification error [31,32]. Models fitted with 1–8 classes based on ten types of daily activities were compared by the AIC, BIC, and nBIC to select the optimal number of latent classes. The model fit improved as the class number increased, but the incremental improvement was comparatively smaller after four and five classes (the fit indices were as follows for models with 4, 5, 6, 7, and 8 classes, respectively: AIC: 111,214.4, 110,854.9, 110,632.6, 110,510.2, and 110,427.2; BIC: 111,532.7, 111,254.6, 111,113.6, 111,072.7, and 111,071.1; and nBIC: 111,396.0, 111,083.0, 110,907.0, 110,831.2, and 110,794.6; see Supplementary Figure S1 for details). Additionally, entropy (0.611 and 0.624, respectively, for the 4- and 5-class models) fell below 0.6 beginning with the 6-class model, and the two classes in the 6-class model had almost the same pattern, which was similar to that of the *Physical-active* class in the 5-class model (as discussed in the Results section). Considering its simplicity and interpretability, we selected the 5-class model because it identifies an additional meaningful latent class compared to the 4-class model and merges two similar classes in the 6-class model.

Demographic differences in latent classes were examined. The most likely class was adjusted by the inclusive classify–analyze approach [33] and then treated as a latent class variable in the subsequent analysis. This inclusive classify–analyze approach can reduce the attenuation of the association between the latent class and cognitive abilities caused by the standard 3-step approach [33]. Specifically, in the inclusive approach, posterior probabilities were generated from a latent class model with cognitive scores included as covariates; then, latent class membership was treated as a known variable according to the most likely class. Further, using SPSS 20, demographic differences across the latent classes were examined by ANOVA. The associations between the latent class and cognitive abilities were further examined by a linear regression analysis after dividing the sample into three age groups (less than 80 years old (the young-old group), *n* = 4042; 80–99.5 years old (the old-old group), *n* = 6595; and more than 100 years old (the oldest-old group), *n* = 1456) according to a previous study [22].

The regression models were built stepwise. First, the crude model (M0), including only the latent class variable, and Model 1 (M1), including only demographic variables, were built. Second, in Model 2 (M2), both the demographic variables and the latent classes were included. Third, in Model 3 (M3), interactions between sex and latent classes were further added based on M2 to examine sex differences in the association between activity engagement and cognition.

## 3. Results

### 3.1. Characteristics of the General Population and Sex Differences

Results in Table 1 show sex differences in demographic characteristics, cognitive abilities, and activity engagement. As expected, male participants were younger, more educated, and more likely to hold professional/administrative jobs. In addition, males were less likely to live alone and had fewer disabilities related to activities of daily living (ADLs), higher cognitive abilities, and a higher prevalence of engagement in almost all activities, except for housework and raising livestock.

### 3.2. Latent Classes of Activity Engagement

**Figure 1:**
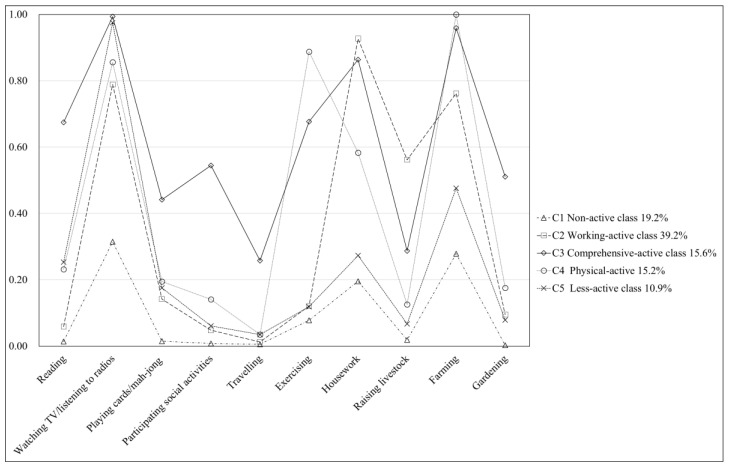
Conditional probability of ten types of activity engagement for the latent classes. Class 1 = Non-active class, Class 2 = Working-active class, Class 3 = Comprehensive-active class, Class 4 = Physical-active, Class 5 = Less-active class.

Figure 1 shows the conditional probability of the ten activities for each subgroup. Class 1 (19.2% of the sample) had a low probability for all activities; this class was thus labeled the Non-active class. All the other latent classes had a high probability of watching TV/listening to the radio. In addition to demonstrating high engagement with public media, Class 2 (39.2%) was characterized by a high probability of doing housework (the highest among all the classes), farming, and raising livestock (mainly for livelihood and less for entertaining) and was thus labeled the Working-active class. Class 3 (15.6%) was characterized by a high probability of engagement in all activities and was thus labeled the Comprehensive-active class. Class 4 (15.2%) was mainly active in farming and exercising (the highest among all the classes), followed by doing housework, all of which are more physically demanding activities. Thus, this class was labeled the Physical-active class. Class 5 (10.9%) was engaged in only entertainment through public media and was moderately engaged in farming; thus, this class was labeled the Less-active class.

### 3.3. Characteristics of the Latent Classes

As demonstrated in Table 2, demographic differences were observed across the latent classes. Specifically, higher proportions of males were present in the Comprehensive-active and the Less-active classes, while higher proportions of females were present in the Non-active and Working-active classes. With respect to age, the Non-active class had the oldest individuals, followed by the Less-active class, the Physical-active class, the Working-active class, and the Comprehensive-active class. Additionally, among all of the classes, the Non-active class reported the worst health and the most severe ADL-related disabilities, while the Comprehensive-active class reported the best health status. Moreover, the Comprehensive-active class had the highest SES with regard to urban living, years of education (schooling), and professional/administrative jobs; in contrast, the Non-active and the Working-active classes had the lowest SES. The Working-active class had the highest number of participants living alone, followed by the Physical-active class, the Non-active and Comprehensive-active classes, and finally, the Less-active class.

### 3.4. Associations of Demographic Factors and Latent Class with Cognitive Ability

Supplementary Table S1 shows the model fit of the regression model of three age groups (less than 80 years old, the young-old group; 80–99.5 years old, the old-old group; and above 100 years old, the oldest-old group) based on a previous study [22]. When the demographic variables (i.e., age, sex, living alone, SES, ADLs, and health) and latent classes were included in the regression model (M2) or the class × sex interaction was further added to M3, the model had higher *R*^2^ values than M0 (with only the latent classes) and M1 (with only the demographic variables) in all cognitive scores across all age groups. Although inclusion of the sex × class interaction yielded a limited increase in *R*^2^ in some models, we used M3 as the final model based on the sex differences found in previous literature and the significant interactions found in the current analysis.

### 3.5. Demographic Factors

As shown in Table 3, for all age groups, almost all cognitive abilities were negatively associated with age (*B*s = −0.08~−0.001, *p*s < 0.01; however, *B* = 0.00, *p =* 0.06 for language ability and *B*s = −0.06~0.00, *ns* for general cognition and orientation and calculation abilities in the oldest-old group) and ADL-related disabilities (*B*s = −0.12~−0.01, *p*s < 0.05; however, *B*s = −0.003 and −0.004, *ns* for calculation and recall abilities in the young-old group, and *B* = −0.07, *ns* for general cognition in the oldest-old group) and positively associated with health status (*B*s = 0.004~0.48, *p*s < 0.05; however, *B* = 0.01, *n*s for calculation ability in the oldest-old group), which may reveal the ageing effect. For the young-old group, males had higher scores for calculation ability (*B* = 0.12, *p* < 0.001) but lower scores for orientation ability (*B* = −0.04, *p* < 0.001). For the old-old group, males scored higher for general cognition as well as calculation ability (*B*s = 0.54 and 0.11, respectively; *p*s < 0.01). In the oldest-old group, males had a higher general cognition (*B* = 1.06, *p* < 0.01). The number of years of education was positively associated with all cognitive abilities in the young-old and old-old groups (*B*s = 0.001~0.10, respectively; *p*s < 0.05), but not in the oldest-old group (*B*s = −0.03~0.003, *ns*). Living in urban areas was associated with higher scores for calculation and language abilities in the young-old group (*B*s = 0.02 and 0.01, *p*s < 0.05) but lower scores for orientation ability in the old-old and the oldest-old groups (*B*s = −0.01 and −0.03, respectively; *p*s < 0.01). Performing professional/administration jobs was related only to general cognition in the old-old group (*B* = 0.35, *p* < 0.05). Living alone was positively related to orientation and language abilities in the old-old group (*B*s = 0.01, respectively; *p*s < 0.05) and positively related to language ability in the oldest-old group (*B* = 0.04, *p* < 0.01). After controlling for all demographic variables, the association between the latent class and cognition and the associated sex differences across age ranges were found as follows.

### 3.6. Associations between the Latent Class and Cognitive Ability across Age Ranges

Compared with the Non-active individuals (serving as the reference class), the other four classes of activity engagement scored significantly higher for general cognition across all age groups (*B*s = 0.76~2.41, *p*s < 0.05; however, *B* = 0.66, *p =* 0.06 for the Less-active class in the oldest-old group). In terms of calculation ability, most of the active classes scored higher than the Non-active class among the young-old and the old-old groups (*B*s = 0.05~0.13, *p*s < 0.01; however, *B* = 0.04, *ns* for the Less-active class in the young-old group). Particularly in the old-old group, the Comprehensive-active and Working-active classes scored higher for recall ability (*B*s = 0.12 and 0.03, respectively; *p*s < 0.05) and the Comprehensive-active and Physical-active classes scored higher on orientation (*B*s = 0.02 and 0.01, respectively; *p*s < 0.05) and language abilities (*B*s = 0.03 and 0.02, respectively; *p*s < 0.05). Notably, the Working-active and Less-active classes had a slightly lower orientation ability than the Non-active class in the young-old group (*B*s = −0.02 and −0.03, respectively; *p*s < 0.05), and the Comprehensive-active class had a slightly lower language ability than the Non-active class in the oldest-old group (*B* = −0.10, *p* < 0.01).

Additionally, the differences in the individuals’ cognitive abilities across the active latent classes were especially obvious in the old-old group. Specifically, as shown in Table 3 (see Supplementary Tables S2–S16 for M0~M3 model results of all the cognitive outcomes), the unstandardized regression coefficient CIs of general cognitive and recall abilities in the Comprehensive-active class were [1.62, 2.55] and [0.07, 0.17], while those in the Working-active class ([0.58, 1.11] and [0.0004, 0.066]), Physical-active class ([0.73, 1.45] and [−0.01, 0.07]), and Less-active class ([0.38, 1.14] and [−0.04, 0.06]) were lower and overlapped with each other, demonstrating that the Comprehensive-active class scored higher than the other three active classes. The regression coefficient CIs of calculation and language abilities were [0.08, 0.17] and [0.01, 0.05] in the Comprehensive-active class, while those in the Working-active class ([0.02, 0.07] and [−0.02, 0.01]) were lower, demonstrating that the Comprehensive-active class scored higher than the *Working-active* class in calculation and language abilities.

### 3.7. Sex Differences in the Associations between the Latent Class and Cognitive Ability

Furthermore, significant sex × class interactions revealed sex differences in the associations between the latent classes and cognitive abilities in some age groups. In the young-old group, for orientation ability, males in all the active classes scored higher than males in the Non-active class, while the opposite pattern was observed for females (*B*s = 0.04~0.07 for the interaction, *ps* < 0.001). In addition, compared with females, males in the Working-active, Comprehensive-active, and Physical-active classes versus the Non-active class had a smaller increase in calculation ability (*B*s = −0.11~−0.07 for the interaction, *p*s < 0.05). In the old-old group, compared with females, males in the Comprehensive-active class versus the Non-active class had a smaller increase in general cognitive and calculation abilities (*B*s = −0.64 and −0.06 for the interaction, *p*s < 0.05).

## 4. Discussion

In the present study, five classes emerged based on an LCA of ten activities performed by older Chinese adults: the Non-active class, Working-active class, Comprehensive-active class, Physical-active class, and Less-active class. Compared with Non-active older individuals, the individuals in the other classes with a higher probability of engaging in various activities (especially the Comprehensive-active class) generally had higher cognitive abilities. However, the associations between being in the classes engaging in certain activities or the Non-active class and cognitive abilities were not always positive across the various age ranges. The differences among the active latent classes were especially obvious in the old-old group as follows: The Comprehensive-active class had higher general cognitive and recall abilities than the other three active classes and higher calculation and language abilities than the Working-active class. Additionally, in the young-old group, the association between being in the active latent classes and orientation ability was positive in the males but negative in the females, while the males exhibited a weaker association between the latent classes and calculation ability; the males also exhibited a weaker association between the latent classes and general cognitive and calculation abilities in the old-old group.

In general, the identified classes were similar to those in studies of elderly in the U.S. Specifically, the Non-active and Comprehensive-active classes were similar to the Low- and High-Activity classes found in previous studies, respectively [14,16,17]. Between these two high and low extremes, we did not find a class of moderate engagement in all activities; instead, we found three classes with varying patterns of activity engagement. The Working-active class was also similar to a class identified in U.S. samples [14,17], although some indicators of activity differed (e.g., farming versus employment/computer use) due to differences in occupation structure. The Physical-active class was also identified in the U.S. samples and was mainly characterized by more involvement in playing sports or physically demanding activities [14,16]. The Less-active class shares some similarity with the Passive Leisure class [17], members of whom only engage in activities necessary to personal life.

The identified classes have different demographic characteristics. Consistent with the previous findings [14,16,17], we found that the Non-active class had the lowest SES and worst health, whereas these factors were most favorable in the Comprehensive-active class. The individuals in the Working-active class, which was characterized by relatively higher proportions of females and younger individuals, were more likely to live alone, were less likely to own an urban residence and hold professional/administrative occupations, had the second lowest number of years of education, and exhibited higher participation in activities necessary for daily life but participated less in relaxing or entertaining activities. Notably, the Working group among the U.S. sample may transition to the group with low activity after retirement, which indicates the necessity of building programs to maximize activity engagement before retirement [14]. The Physical-active class had higher probabilities of owning an urban residence, similar to the findings of a previous study [14]. In addition, they had relatively higher education, professional/administrative occupations, better health status, and moderate age. The individuals in the Less-active class, which was characterized by relatively older age, a higher proportion of males, moderate SES based on the number of years of education, moderate probability of owning an urban residence and holding a professional/administrative occupation, and relatively worse health status, were the least likely to live alone and had only a medium probability of engaging in farming in addition to media entertainment.

After adjusting for the confounders (i.e., age, sex, health, SES, and living alone), the regression analysis further demonstrated associations between certain activity engagement classes and cognitive abilities among older Chinese adults across different age ranges. Generally, similar to previous research findings [16,17], membership in the four active classes versus the Non-active class was mostly associated with higher levels of cognitive abilities. This association is consistent with the findings of previous studies showing a positive association between activity engagement and cognitive abilities among older adults [6,8,9,10,34], and this association has been related to both the diversity and prevalence of engagement [35,36,37]. In contrast, the Non-active class, which had the oldest age, showed possible declines, and the activity engagement of individuals in this class was therefore limited.

Furthermore, the differences in the individuals’ cognitive abilities across the active latent classes were especially obvious in the old-old group, as follows: the Comprehensive-active class had higher general cognitive and recall abilities than the other three active classes and higher calculation and language abilities than the Working-active class. Unexpectedly, compared to the Non-active class, the Working-active and Less-active classes had a lower orientation ability in the young-old old group; the class engaging in comprehensive activities had a lower language ability than the other classes in the oldest-old group. These associations reveal that not all patterns of being active in daily life are associated with a better cognitive status among older individuals. Moreover, the associations between activity engagement and cognitive abilities seem to decrease as individuals grow extremely old.

Consistent with the findings of previous studies [27,38], the current study found generally higher cognitive abilities among the males. In addition, we found a higher percentage of males in the Comprehensive-active class and Less-active class, while a higher percentage of females was found in the Non-active class and the Working-active class, which is the opposite of the findings for the U.S. sample [14]. In addition to a younger age among the males, the sex differences in cognitive abilities and activity engagement may be due to differences in social status and roles in the participants’ earlier lives. For example, males with a more advantageous social status as well as better nutrition and education spent more time working in various industries, while females focused only on farming and supporting the family indoors through activities such as housework and child-rearing. With relatively better education and social networks, males are often more extroverted and open to diverse activities, including leisure activities, during their retired life [39,40,41,42]. Previous studies [27] have shown that social networks and participation in leisure activities partially explained the sex differences in cognitive impairment.

Among the young-old and old-old groups, we also found sex differences in the association between activity engagement and different cognitive abilities after controlling for the main effect of sex and other demographic variables. Specifically, in the young-old group, males in active classes had a higher orientation ability than did males in the Non-active class; in contrast, females in active classes had a lower orientation ability than did females in the Non-active class. However, when comparing individuals of some active classes versus Non-active individuals, males showed a smaller increase in general cognition (in the old-old group) and calculation ability (in both age groups) compared to females. Taken together, we propose that activity engagement is a partial mediator of the relationship between sex and cognitive ability and that the links between activity engagement and cognitive ability is moderated by sex. Such a model that includes more complex relational paths should be further examined in a longitudinal study.

### Study Limitations

Several limitations of this study are worth noting. First, future research should consider the frequency of activity engagement and the level of satisfaction that older adults have with activities to establish a more elaborate classification of the ageing population. Second, the cognitive abilities and activity engagement were assessed simultaneously, and the results only revealed the associations between activity engagement and cognitive abilities. The effect of activity engagement should be examined in future longitudinal studies. Third, associations of cognitive function with nutritional status and dietary pattern have been well-documented in previous epidemiological and observational studies [43,44,45,46,47], but these nutritional factors were not collected within Chinese Longitudinal Healthy Longevity Survey (CLHLS) data. Thus, a similar topic should be further studied with an inclusion of nutritional factors. In addition, individuals with a more active style of life (or highly scholarly educated persons) tend to pay more attention to a better and balanced diet including more healthy food; thus, it still remains unclear whether cognitive benefit is associated with activity engagement alone or combined effects (higher activity engagement, better nutritional level, and greater academic attainment).

## 5. Practical Implications

The current study has some implications for future research and practice in this field. By using LCA, the current study replicated five types of activity engagement found in the previous literature [14,16,17], thus strengthening the call for investigating multiple-activity engagement of the elderly via a person-centered approach. This approach not only helps document some specific groups under various conceptualizations, especially for those who do not achieve “active aging” [16,17], but also extends our understanding of the activity–cognition link considering high-order interaction. For practice, the classes identified based on a nationally representative sample of older Chinese adults provide important guidance for the design of policies and programs for the elderly on the Chinese mainland. In particular, the relatively higher proportions of the *Non-active* class and *Working-active* class may imply greater challenges for healthy aging and the need for related programs in China [14]. Furthermore, based on the diverse activity–cognition links, culture-specific programs (traditional Chinese exercises) [48,49,50] should be tailored to subgroups of different ages and genders by providing different training or activity modules based on their related dimensions of cognitive decline.

## Figures and Tables

**Table 1:** Descriptive Statistics of the Investigated Variables.

Variable	Total			Male			Female			Comparison (*t*/χ^2^)
	*M*	*SD*	Range	*M*	*SD*	Range	*M*	*SD*	Range	
Validated age	84.60	10.96	65~116	82.81	10.16	65~110	86.18	11.38	65~116	M < F (−17.20 ***)
Living alone (%)	16.70	—	—	15.07	—	—	18.12	—	—	M < F (20.20 ***)
Urban residence (%)	20.95	—	—	21.19	—	—	20.74	—	—	M > F (0.37)
Years of education	2.32	3.49	0~25	3.75	3.89	0~25	1.06	2.50	0~18	M > F (44.55 ***)
Professional/administration occupation (%)	8.41	—	—	14.18	—	—	3.34	—	—	M > F (458.78 ***)
ADL-related disability	6.43	1.46	6~18	6.30	1.17	6~18	6.56	1.66	6~18	M < F (−10.06 ***)
Subjective health	3.49	0.91	1~5	3.53	0.91	1~5	3.46	0.91	1~5	M > F (4.14)
General cognition	27.06	3.17	18.54~30	27.65	2.79	19.50~30	26.54	3.39	18.54~30	M > F (19.68 ***)
Orientation	0.97	0.10	0~1	0.98	0.08	0~1	0.96	0.12	0~1	M > F (11.90 ***)
Calculation	0.86	0.27	0~1	0.92	0.21	0~1	0.80	0.31	0~1	M > F (22.90 ***)
Recall	0.80	0.34	0~1	0.82	0.32	0~1	0.77	0.35	0~1	M > F (8.49 ***)
Language	0.95	0.13	0~1	0.96	0.12	0~1	0.94	0.14	0~1	M > F (9.20 ***)
Reading (%)	20.57	—	—	33.32	—	—	9.39	—	—	M > F (1055.55 ***)
Watching TV/listening to radios (%)	75.82	—	—	82.40	—	—	70.05	—	—	M > F (250.29 ***)
Playing cards/mah-jong (%)	17.82	—	—	22.49	—	—	13.73	—	—	M > F (157.52 ***)
Participating social activities (%)	13.90	—	—	17.39	—	—	10.85	—	—	M > F (107.61 ***)
Travelling (%)	5.92	—	—	6.87	—	—	5.08	—	—	M > F (17.50 ***)
Exercising (%)	31.63	—	—	38.56	—	—	25.56	—	—	M > F (235.34 ***)
Housework (%)	62.20	—	—	59.15	—	—	64.87	—	—	M < F (41.86 ***)
Raising livestock (%)	26.92	—	—	27.11	—	—	26.76	—	—	M > F (0.18)
Farming (%)	68.82	—	—	73.18	—	—	65.01	—	—	M > F (93.52 ***)
Gardening (%)	15.54	—	—	18.40	—	—	13.03	—	—	M > F (65.96 ***)

*Note*. *** *p* < 0.001. Proportion (yes %) and χ^2^ test were applied for binary variables, and *t* test was applied for continuous variables.

**Table 2:** Characteristics of Latent Class and Pair-wise Comparisons.

Variable	C1 Non-Active		C2 Working-Active		C3 Comprehensive-Active		C4 Physical-Active		C5 Less-Active		Pair-Wise Comparison (*F*/χ^2^)
	*M* (*SD*)	Range	*M* (*SD*)	Range	*M* (*SD*)	Range	*M* (*SD*)	Range	*M* (*SD*)	Range	
Validated Age	92.64 (8.51)	65~116	82.28 (10.31)	65~110	77.77 (9.56)	65~106	84.48 (10.38)	65~108	88.72 (9.73)	65~114	C3 < C2 < C4 < C5 < C1 (742.59 ***)
Male (%)	34.35	—	38.99	—	64.69	—	54.12	—	59.98.	—	C1 < C2 < C4 < C5 < C3 (2319.97 ***)
Living alone (%)	14.88	—	20.95	—	13.66	—	18.06	—	7.14	—	C5 < C3, C1 < C4 < C2 (217.94 ***)
Urban residence (%)	15.78	—	7.91	—	41.77	—	35.57	—	26.50	—	C2 < C1 < C5 < C4 < C3 (937.92 ***)
Years of education	1.16 (2.48)	0~17	1.49 (2.61)	0~25	5.19 (4.34)	0~25	2.66 (3.60)	0~22	2.73 (3.79)	0~23	C1 < C2 < C4, C5 < C3 (541.69 ***)
Professional/administration occupation (%)	3.66	—	2.70	—	24.70	—	11.47	—	9.57	—	C2, C1 < C5 < C4 < C3 (1065.46 ***)
ADL-related disability	7.55 (2.65)	6~18	6.06 (0.40)	6~16	6.07 (0.45)	6~14	6.22 (0.83)	6~16	6.62 (1.51)	6~18	C2, C3 < C4 < C5 < C1 (548.67 ***)
Subjective health	3.28 (0.96)	1~5	3.47 (0.88)	1~5	3.72 (0.88)	1~5	3.59 (0.88)	1~5	3.46 (0.91)	1~5	C1 < C5, C2 < C4 < C3 (67.16 ***)
General cognition	25.26 (3.51)	18.54~30	27.31 (2.99)	18.54~30	28.64 (2.08)	18.63~30	27.38 (2.88)	18.70~30	26.66 (3.31)	18.54~30	C1 < C5 < C2, C4 < C3 (360.26 ***)
Orientation	0.94 (0.14)	0~1	0.97 (0.10)	0~1	0.99 (0.05)	0.40~1	0.98 (0.09)	0~1	0.96 (0.11)	0~1	C1 < C5 < C2, C4 < C3 (75.47 ***)
Calculation	0.75 (0.34)	0~1	0.86 (0.27)	0~1	0.95 (0.16)	0~1	0.87 (0.26)	0~1	0.85 (0.27)	0~1	C1 < C5, C2, C4 < C3 (132.09 ***)
Recall	0.71 (0.38)	0~1	0.80 (0.33)	0~1	0.89 (0.26)	0~1	0.79 (0.34)	0~1	0.77 (0.35)	0~1	C1 < C5, C4, C2 < C3 (67.00 ***)
Language	0.92 (0.17)	0~1	0.95 (0.13)	0~1	0.98 (0.08)			0.17~1	0.97 (0.09)	0~1	C1< C5 < C2 < C4 < C3 (75.52 ***)

*Note*: *** *p* < 0.001. Proportion (yes%) and χ^2^ test were applied for binary variables, and *F* test was applied for continuous variables.

**Table 3:** Unstandardized Beta Coefficients and 95% CI of Regression Model 3 for Cognitive Abilities.

Age Group	Variable	General Cognition	Orientation	Calculation	Recall	Language
The young-elderly group (below 80) *N* = 4042, MMSE general cognition ranging 18.57~30.00	Age	−0.07 ***	−0.001 ***	−0.004 ***	−0.01 ***	−0.001 **
		[−0.09, −0.05]	[−0.002, −0.001]	[−0.006, −0.003]	[−0.009, −0.004]	[−0.002, −0.0004]
	Male	0.11	−0.04 ***	0.12 ***	−0.04	−0.02
		[−0.56, 0.78]	[−0.06, −0.02]	[0.06, 0.18]	[−0.12, 0.06]	[−0.04, 0.01]
	Living alone	−0.18 ^†^	0.001	0.0004	−0.003	−0.004
		[−0.37, 0.02]	[−0.01, 0.01]	[−0.02, 0.02]	[−0.03, 0.02]	[−0.011, 0.003]
	Urban residence	−0.02	−0.001	0.02 *	−0.01	0.01 *
		[−0.20, 0.16]	[−0.01, 0.01]	[0.001, 0.033]	[−0.03, 0.02]	[0.0004, 0.014]
	Years of education	0.07 ***	0.001 *	0.01 ***	0.01 ***	0.001 **
		[0.05, 0.09]	[0.00008, 0.001]	[0.003, 0.007]	[0.003, 0.009]	[0.0004, 0.002]
	Professional/administration occupation	−0.002	0.0004	−0.003	0.002	−0.001
		[−0.25, 0.24]	[−0.01, 0.01]	[−0.02, 0.02]	[−0.03, 0.03]	[−0.01, 0.01]
	ADL-related disability	−0.11 *	−0.01 ***	−0.003	−0.004	−0.01 **
		[−0.21, −0.01]	[−0.01, −0.01]	[−0.01, 0.01]	[−0.02, 0.01]	[−0.009, −0.002]
	Subjective health	0.25 ***	0.004 ***	0.01 ***	0.02 ***	0.01 **
		[0.18, 0.33]	[0.002, 0.006]	[0.01, 0.02]	[0.02, 0.03]	[0.002, 0.007]
	C2	1.10 ***	−0.02 *	0.10 ***	−0.003	0.004
		[0.56, 1.64]	[−0.04, −0.002]	[0.05, 0.15]	[−0.08, 0.07]	[−0.02, 0.02]
	C3	1.42 ***	−0.01	0.13 ***	0.01	0.02
		[0.85, 1.99]	[−0.03, 0.01]	[0.08, 0.18]	[−0.07, 0.08]	[−0.01, 0.04]
	C4	1.12 ***	−0.02 ^†^	0.09 **	−0.02	0.01
		[0.54, 1.70]	[−0.03, 0.002]	[0.04, 0.14]	[−0.10, 0.06]	[−0.01, 0.03]
	C5	0.97 *	−0.03 *	0.04	−0.02	−0.01
		[0.14, 1.79]	[−0.06, −0.01]	[−0.04, 0.11]	[−0.13, 0.09]	[−0.04, 0.02]
	C2×Male	0.26	0.05 ***	−0.07 *	0.04	0.02 ^†^
		[−0.44, 0.95]	[0.03, 0.07]	[−0.13, −0.01]	[−0.05, 0.13]	[−0.001, 0.049]
	C3×Male	0.15	0.04 ***	−0.11 **	0.04	0.02
		[−0.56, 0.86]	[0.02, 0.07]	[−0.17, −0.05]	[−0.06, 0.13]	[−0.01, 0.04]
	C4×Male	0.09	0.05 ***	−0.08 *	0.03	0.02
		[−0.66, 0.84]	[0.03, 0.07]	[−0.15, −0.01]	[−0.07, 0.13]	[−0.01, 0.05]
	C5×Male	0.29	0.07 ***	−0.001	0.06	0.03 ^†^
		[−0.69, 1.26]	[0.04, 0.10]	[−0.09, 0.08]	[−0.07, 0.19]	[−0.003, 0.068]
The old-old group (80–99.5) *N* = 6595, MMSE general cognition ranging 18.54~30.00	Age	−0.08 ***	−0.002 ***	−0.004 ***	−0.002 **	−0.002 ***
		[−0.09, −0.06]	[−0.002, −0.001]	[−0.006, 0.003]	[−0.004, −0.001]	[−0.003, −0.001]
	Male	0.54 **	0.01	0.11 ***	0.04 ^†^	−0.001
		[0.22, 0.86]	[−0.002, 0.021]	[0.08, 0.14]	[−0.001, 0.08]	[−0.02, 0.01]
	Living alone	−0.07	0.01 *	0.02 ^†^	0.01	0.01 **
		[−0.26, 0.11]	[0.001, 0.02]	[−0.001, 0.035]	[−0.01, 0.04]	[0.01, 0.02]
	Urban residence	−0.14	−0.01 ***	0.01	−0.02 ^†^	0.01 ^†^
		[−0.33, 0.06]	[−0.02, −0.01]	[−0.01, 0.02]	[−0.045, 0.002]	[−0.001, 0.017]
	Years of education	0.10 ***	0.002 ***	0.01 ***	0.01 ***	0.003 ***
		[0.07, 0.13]	[0.001, 0.003]	[0.004, 0.01]	[0.004, 0.011]	[0.002, 0.004]
	Professional/administration occupation	0.35 *	0.0004	0.004	0.03 ^†^	0.01 ^†^
		[0.03, 0.68]	[−0.01, 0.01]	[−0.03, 0.04]	[−0.01, 0.07]	[−0.002, 0.028]
	ADL-related disability	−0.12 ***	−0.01 ***	−0.01 **	−0.01 *	−0.01 ***
		[−0.18, −0.07]	[−0.01, −0.01]	[−0.014, −0.003]	[−0.014, 0.0002]	[−0.008, −0.003]
	Subjective health	0.29 ***	0.01 ***	0.02 ***	0.03 ***	0.01 ***
		[0.21, 0.37]	[0.01, 0.01]	[0.01, 0.02]	[0.02, 0.04]	[0.01, 0.02]
	C2	0.85 ***	0.01	0.05 **	0.03 *	−0.01
		[0.58, 1.11]	[−0.01, 0.02]	[0.02, 0.07]	[0.0004, 0.066]	[−0.02, 0.01]
	C3	2.09 ***	0.02 *	0.13 ***	0.12 ***	0.03 *
		[1.62, 2.55]	[0.003, 0.04]	[0.08, 0.17]	[0.07, 0.17]	[0.01, 0.05]
	C4	1.09 ***	0.01 *	0.05 **	0.03	0.02 *
		[0.73, 1.45]	[0.001, 0.03]	[0.02, 0.09]	[−0.01, 0.07]	[0.004, 0.038]
	C5	0.76 ***	0.01 ^†^	0.08 ***	0.01	0.002
		[0.38, 1.14]	[0.00003, 0.03]	[0.04, 0.12]	[−0.04, 0.06]	[−0.02, 0.02]
	C2 × Male	−0.01	0.004	−0.002	−0.02	0.01
		[−0.41, 0.4]	[−0.01, 0.02]	[−0.04, 0.04]	[−0.07, 0.03]	[−0.01, 0.03]
	C3 × Male	−0.64 *	−0.000004	−0.06 *	−0.04	−0.01
		[−1.22, −0.07]	[−0.02, 0.02]	[−0.12, −0.01]	[−0.11, 0.03]	[−0.04, 0.02]
	C4 × Male	−0.07	0.01	−0.01	−0.02	0.01
		[−0.57, 0.42]	[−0.01, 0.02]	[−0.05, 0.04]	[−0.08, 0.04]	[−0.01, 0.03]
	C5 × Male	−0.22	−0.002	−0.04	0.01	0.01
		[−0.74, 0.3]	[−0.02, 0.02]	[−0.09, 0.01]	[−0.05, 0.07]	[−0.02, 0.03]
The oldest-old group (above 100) *N* = 1456, MMSE general cognition ranging 18.54~30.00	Age	−0.06	0.002	0.002	−0.02 **	−0.004 ^†^
		[−0.14, 0.03]	[−0.01, −0.002]	[−0.01, 0.01]	[−0.03, −0.01]	[−0.008, 0.0002]
	Male	1.06 **	0.02	0.08 ^†^	0.02	0.01
		[0.31, 1.81]	[−0.01, 0.05]	[−0.004, 0.154]	[−0.07, 0.10]	[−0.03, 0.05]
	Living alone	−0.12	0.01	0.04	0.03	0.04 **
		[−0.73, 0.49]	[−0.01, 0.04]	[−0.03, 0.10]	[−0.04, 0.10]	[0.01, 0.07]
	Urban residence	0.37	−0.03 **	−0.001	0.004	0.01
		[−0.09, 0.83]	[−0.04, −0.01]	[−0.05, 0.05]	[−0.05, 0.06]	[−0.01, 0.03]
	Years of education	−0.03	0.0001	0.003	−0.01	0.001
		[−0.12, 0.07]	[−0.004, 0.004]	[−0.01, 0.01]	[−0.017, 0.004]	[−0.004, 0.005]
	Professional/administration occupation	1.11 ^†^	0.02	0.10 ^†^	0.12 ^†^	0.04
		[−0.07, 2.28]	[−0.03, 0.07]	[−0.01, 0.22]	[−0.01,0.25]	[−0.02, 0.10]
	ADL-related disability	−0.07	−0.01 **	−0.02 **	−0.02 ***	−0.01 **
		[−0.16, 0.02]	[−0.008, −0.001]	[−0.03, −0.01]	[−0.03, −0.01]	[−0.012, −0.003]
	Subjective health	0.48 ***	0.02 **	0.01	0.03 *	0.02 **
		[0.25, 0.70]	[0.01, 0.02]	[−0.01, 0.04]	[0.003, 0.055]	[0.004, 0.026]
	C2	1.51 ***	0.02	0.04	0.05 ^†^	−0.004
		[0.98, 2.04]	[−0.01, 0.04]	[−0.02, 0.10]	[−0.01, 0.11]	[−0.03, 0.02]
	C3	2.41 **	0.04	−0.02	0.11	−0.10 **
		[1.04, 3.79]	[−0.02, 0.09]	[−0.16, 0.11]	[−0.04, 0.26]	[−0.17, −0.04]
	C4	1.03 **	0.003	0.06	−0.002	0.001
		[0.28, 1.78]	[−0.03, 0.03]	[−0.02, 0.14]	[−0.09, 0.08]	[−0.04, 0.04]
	C5	0.66 ^†^	0.002	0.003	0.05	−0.001
		[−0.03, 1.34]	[−0.03, 0.03]	[−0.07, 0.07]	[−0.03, 0.12]	[−0.03, 0.03]
	C2 × Male	0.04	−0.01	0.08	−0.03	0.02
		[−1.23, 1.30]	[−0.06, 0.04]	[−0.05, 0.21]	[−0.17, 0.11]	[−0.05, 0.08]
	C3 × Male	−0.31	−0.01	0.10	−0.05	0.10 ^†^
		[−2.32, 1.70]	[−0.09, 0.07]	[−0.10, 0.30]	[−0.26, 0.17]	[−0.001, 0.192]
	C4 × Male	−0.62	0.003	0.01	0.03	0.003
		[−1.88, 0.64]	[−0.05, 0.05]	[−0.13, 0.14]	[−0.11, 0.18]	[−0.06, 0.06]
	C5 × Male	−0.71	0.01	0.01	−0.03	−0.02
		[−1.94, 0.52]	[−0.03, 0.06]	[−0.12, 0.14]	[−0.17, 0.11]	[−0.08, 0.04]

*Note*. ^†^*p* < 0.10, * *p* < 0.05, ** *p* < 0.01, *** *p* < 0.001. C1 Non-active class, C2 Working-active class, C3 Comprehensive-active class, C4 Physical-active class, C5 Less-active class.

## Supplementary Materials

The following are available online at https://www.mdpi.com/2076-3425/10/12/967/s1, Table S1: Model fits (*R*^2^) of regression models for cognitive abilities., Table S2: Regression models of general cognition among the young-old group, Table S3: Regression models of orientation ability among the young-old group, Table S4: Regression models of calculation ability among the young-old group, Table S5: Regression models of recall ability among the young-old group, Table S6: Regression models of language ability among the young-old group, Table S7: Regression models of general cognition among the old-old group, Table S8: Regression models of orientation ability among the old-old group, Table S9: Regression models of calculation ability among the old-old group, Table S10: Regression models of recall ability among the old-old group, Table S11: Regression models of language ability among the old-old group, Table S12: Regression models of general cognition among the oldest-old group, Table S13: Regression models of orientation ability among the oldest-old group, Table S14: Regression models of calculation ability among the oldest-old group, Table S15: Regression models of recall ability among the oldest-old group, Table S16: Regression models of language ability among the oldest-old group, Figure S1: Information criteria for 1–8 class model.
