# Supplementary material for: Cognitive Benefits of Activity Engagement among 12,093 Adults Aged over 65 Years
Source: Brain Sci. 2020 Dec 10;10(12):967. doi: 10.3390/brainsci10120967 (PMC7764821; doi:10.3390/brainsci10120967)
Supplement: Supplementary file 1 [file brainsci-10-00967-s001.pdf]

Supplementary Table S1. Model fits (R^2) of regression models for cognitive abilities.

Model	The young-elderly group (below 80 years)					The old-old group (80-99.5 years)					The oldest-old group (above 100 years)				
	General cognition	Orientation	Calculation	Recall	Language	General cognition	Orientation	Calculation	Recall	Language	General cognition	Orientation	Calculation	Recall	Language
M0	.043	.011	.021	.007	.017	.057	.015	.027	.015	.027	.048	.010	.015	.013	.015
M1	.089	.0.32	.064	.032	.029	.091	.046	.075	.028	.034	.053	.038	.048	.041	.030
M2	.102	.033	.068	.033	.034	.110	.048	.081	.032	.038	.086	.041	.053	.045	.034
M3	.102	.039	.073	.033	.035	.111	.049	.082	.033	.039	.087	.042	.055	.046	.038

Supplementary Table S2. Regression models of general cognition among the young-old group.

Variable	M0			M1			M2			M3		
	β	*SE*	95% CI	β	*SE*	95% CI	β	*SE*	95% CI	β	*SE*	95% CI
Age	—	—	—	-0.07***	0.01	[-0.09,-0.06]	-0.07***	0.01	[-0.09,-0.05]	-0.07***	0.01	[-0.09,-0.05]
Male	—	—	—	0.29***	0.07	[0.15,0.43]	0.30***	0.07	[0.16,0.45]	0.11	0.34	[-0.56,0.78]
Living alone	—	—	—	-0.15	0.10	[-0.35,0.04]	-0.17†	0.10	[-0.36,0.02]	-0.18†	0.10	[-0.37,0.02]
Urban residence	—	—	—	0.07	0.09	[-0.10,0.24]	-0.02	0.09	[-0.20,0.17]	-0.02	0.09	[-0.20,0.16]
Years of education	—	—	—	0.08***	0.01	[0.06,0.10]	0.07***	0.01	[0.05,0.09]	0.07***	0.01	[0.05,0.09]
Professional/administration occupation	—	—	—	0.02	0.12	[-0.22,0.27]	-0.01	0.12	[-0.25,0.23]	0.00	0.13	[-0.25,0.24]
ADL-related disability	—	—	—	-0.23***	0.05	[-0.33,-0.14]	-0.11*	0.05	[-0.21,0]	-0.11*	0.05	[-0.21,-0.01]
Subjective health	—	—	—	0.29***	0.04	[0.22,0.36]	0.25***	0.04	[0.18,0.32]	0.25***	0.04	[0.18,0.33]
C2	1.56***	0.18	[1.21,1.91]	—	—	—	1.24***	0.19	[0.87,1.60]	1.10***	0.28	[0.56,1.64]
C3	2.26***	0.18	[1.90,2.61]	—	—	—	1.50***	0.20	[1.12,1.89]	1.42***	0.29	[0.85,1.99]
C4	1.56***	0.19	[1.19,1.94]	—	—	—	1.18***	0.20	[0.78,1.57]	1.12***	0.30	[0.54,1.70]
C5	1.61***	0.23	[1.17,2.05]	—	—	—	1.15***	0.23	[0.71,1.6]	0.97*	0.42	[0.14,1.79]
C2×Male	—	—	—	—	—	—	—	—	—	0.26	0.35	[-0.44,0.95]
C3×Male	—	—	—	—	—	—	—	—	—	0.15	0.36	[-0.56,0.86]
C4×Male	—	—	—	—	—	—	—	—	—	0.09	0.38	[-0.66,0.84]
C5×Male	—	—	—	—	—	—	—	—	—	0.29	0.50	[-0.69,1.26]

Note. †$p < .10$, *$p < .05$, **$p < .01$, ***$p < .001$. The regression coefficients in Tables 2–16 are unstandardized betas.

Supplementary Table S3. Regression models of orientation ability among the young-old group.

Variable	M0			M1			M2			M3		
	β	*SE*	95% CI	β	*SE*	95% CI	β	*SE*	95% CI	β	*SE*	95% CI
Age	—	—	—	0.00^{***}	0.00	[0,0]	0.00^{***}	0.00	[0,0]	0.00^{***}	0.00	[0,0]
Male	—	—	—	0.01^{***}	0.00	[0,0.01]	0.01^{*}	0.00	[0,0.01]	-0.04^{***}	0.01	[-0.06,-0.02]
Living alone	—	—	—	0.00	0.00	[-0.01,0.01]	0.00	0.00	[-0.01,0.01]	0.00	0.00	[-0.01,0.01]
Urban residence	—	—	—	0.00	0.00	[0,0.01]	0.00	0.00	[-0.01,0.01]	0.00	0.00	[-0.01,0.01]
Years of education	—	—	—	0.00^{*}	0.00	[0,0]	0.00^{*}	0.00	[0,0]	0.00^{*}	0.00	[0,0]
Professional/administration occupation	—	—	—	0.00	0.00	[-0.01,0.01]	0.00	0.00	[-0.01,0.01]	0.00	0.00	[-0.01,0.01]
ADL-related disability	—	—	—	-0.01^{***}	0.00	[-0.01,-0.01]	-0.01^{***}	0.00	[-0.01,-0.01]	-0.01^{***}	0.00	[-0.01,-0.01]
Subjective health	—	—	—	0.00^{***}	0.00	[0,0.01]	0.00^{***}	0.00	[0,0.01]	0.00^{***}	0.00	[0,0.01]
C2	0.02^{***}	0.01	[0.01,0.03]	—	—	—	$0.01^{\dagger}$	0.01	[0,0.02]	-0.02^{*}	0.01	[-0.04,0]
C3	0.03^{***}	0.01	[0.02,0.04]	—	—	—	0.01^{*}	0.01	[0,0.03]	-0.01	0.01	[-0.03,0.01]
C4	0.03^{***}	0.01	[0.02,0.04]	—	—	—	0.01^{*}	0.01	[0,0.02]	$-0.02^{\dagger}$	0.01	[-0.03,0]
C5	0.03^{**}	0.01	[0.01,0.04]	—	—	—	0.01	0.01	[0,0.02]	-0.03^{*}	0.01	[-0.06,-0.01]
C2×Male	—	—	—	—	—	—	—	—	—	0.05^{***}	0.01	[0.03,0.07]
C3×Male	—	—	—	—	—	—	—	—	—	0.04^{***}	0.01	[0.02,0.07]
C4×Male	—	—	—	—	—	—	—	—	—	0.05^{***}	0.01	[0.03,0.07]
C5×Male	—	—	—	—	—	—	—	—	—	0.07^{***}	0.02	[0.04,0.10]

Note. $^{\dagger}p < .10$, $^{*}p < .05$, $^{**}p < .01$, $^{***}p < .001$.

Supplementary Table S4. Regression models of calculation ability among the young-old group.

Variable	M0			M1			M2			M3		
	β	*SE*	95% CI	β	*SE*	95% CI	β	*SE*	95% CI	β	*SE*	95% CI
Age	—	—	—	-0.01***	0.00	[-0.01,0]	0.00***	0.00	[-0.01,0]	0.00***	0.00	[-0.01,0]
Male	—	—	—	0.04***	0.01	[0.03,0.06]	0.04***	0.01	[0.03,0.06]	0.12***	0.03	[0.06,0.18]
Living alone	—	—	—	0.00	0.01	[-0.02,0.02]	0.00	0.01	[-0.02,0.02]	0.00	0.01	[-0.02,0.02]
Urban residence	—	—	—	0.02**	0.01	[0.01,0.04]	0.02*	0.01	[0,0.04]	0.02*	0.01	[0,0.03]
Years of education	—	—	—	0.01***	0.00	[0,0.01]	0.01***	0.00	[0,0.01]	0.01***	0.00	[0,0.01]
Professional/administration occupation	—	—	—	0.00	0.01	[-0.02,0.02]	-0.01	0.01	[-0.03,0.02]	0.00	0.01	[-0.02,0.02]
ADL-related disability	—	—	—	-0.01*	0.00	[-0.02,0]	-0.01	0.01	[-0.01,0]	0.00	0.01	[-0.01,0.01]
Subjective health	—	—	—	0.02***	0.00	[0.01,0.02]	0.01***	0.00	[0.01,0.02]	0.01***	0.00	[0.01,0.02]
C2	0.07***	0.02	[0.03,0.10]	—	—	—	0.06**	0.02	[0.02,0.09]	0.10***	0.03	[0.05,0.15]
C3	0.12***	0.02	[0.08,0.15]	—	—	—	0.07***	0.02	[0.03,0.10]	0.13***	0.03	[0.08,0.18]
C4	0.06***	0.02	[0.03,0.09]	—	—	—	0.04*	0.02	[0,0.07]	0.09**	0.03	[0.04,0.14]
C5	0.08***	0.02	[0.04,0.12]	—	—	—	0.05*	0.02	[0.01,0.09]	0.04	0.04	[-0.04,0.11]
C2×Male	—	—	—	—	—	—	—	—	—	-0.07*	0.03	[-0.13,-0.01]
C3×Male	—	—	—	—	—	—	—	—	—	-0.11**	0.03	[-0.17,-0.05]
C4×Male	—	—	—	—	—	—	—	—	—	-0.08*	0.03	[-0.15,-0.01]
C5×Male	—	—	—	—	—	—	—	—	—	0.00	0.04	[-0.09,0.08]

Note. †*p*< .10, **p*< .05, ***p*< .01, ****p*< .001.

Supplementary Table S5. Regression models of recall ability among the young-old group.

Variable	M0			M1			M2			M3		
	β	*SE*	95% CI	β	*SE*	95% CI	β	*SE*	95% CI	β	*SE*	95% CI
Age	—	—	—	-0.01***	0.00	[-0.01,0.01]	-0.01***	0.00	[-0.01,0]	-0.01***	0.00	[-0.01,0]
Male	—	—	—	0.00	0.01	[-0.02,0.02]	0.00	0.01	[-0.02,0.02]	-0.04	0.05	[-0.12,0.06]
Living alone	—	—	—	0.00	0.01	[-0.03,0.02]	0.00	0.01	[-0.03,0.02]	0.00	0.01	[-0.03,0.02]
Urban residence	—	—	—	-0.01	0.01	[-0.03,0.02]	-0.01	0.01	[-0.03,0.02]	-0.01	0.01	[-0.03,0.02]
Years of education	—	—	—	0.01***	0.00	[0,0.01]	0.01***	0.00	[0,0.01]	0.01***	0.00	[0,0.01]
Professional/administration occupation	—	—	—	0.00	0.02	[-0.03,0.04]	0.00	0.02	[-0.03,0.03]	0.00	0.02	[-0.03,0.03]
ADL-related disability	—	—	—	-0.01	0.01	[-0.02,0.01]	0.00	0.01	[-0.02,0.01]	0.00	0.01	[-0.02,0.01]
Subjective health	—	—	—	0.03***	0.01	[0.02,0.04]	0.02***	0.01***	[0.02,0.03]	0.02***	0.01	[0.02,0.03]
C2	0.04†	0.02	[0,0.09]	—	—	—	0.02	0.03	[-0.03,0.07]	0.00	0.04	[-0.08,0.07]
C3	0.09***	0.02	[0.04,0.13]	—	—	—	0.03	0.03	[-0.02,0.08]	0.01	0.04	[-0.07,0.08]
C4	0.03	0.03	[-0.02,0.08]	—	—	—	0.00	0.03	[-0.06,0.05]	-0.02	0.04	[-0.10,0.06]
C5	0.05†	0.03	[0,0.11]	—	—	—	0.02	0.03	[-0.04,0.08]	-0.02	0.06	[-0.13,0.09]
C2×Male	—	—	—	—	—	—	—	—	—	0.04	0.05	[-0.05,0.13]
C3×Male	—	—	—	—	—	—	—	—	—	0.04	0.05	[-0.06,0.13]
C4×Male	—	—	—	—	—	—	—	—	—	0.03	0.05	[-0.07,0.13]
C5×Male	—	—	—	—	—	—	—	—	—	0.06	0.07	[-0.07,0.19]

Note. $^{\dagger}p < .10$, $^{*}p < .05$, $^{**}p < .01$, $^{***}p < .001$.

Supplementary Table S6. Regression models of language ability among the young-old group.

Variable	M0			M1			M2			M3		
	β	SE	95% CI	β	SE	95% CI	β	SE	95% CI	β	SE	95% CI
Age	—	—	—	0.00***	0.00	[0,0]	0.00**	0.00	[0,0]	0.00**	0.00	[0,0]
Male	—	—	—	0.00†	0.00	[0,0.01]	0.01†	0.00	[0,0.01]	-0.02	0.01	[-0.04,0.01]
Living alone	—	—	—	0.00	0.00	[-0.01,0]	0.00	0.00	[-0.01,0]	0.00	0.00	[-0.01,0]
Urban residence	—	—	—	0.01**	0.00	[0,0.02]	0.01**	0.00	[0,0.01]	0.01*	0.00	[0,0.01]
Years of education	—	—	—	0.00**	0.00	[0,0]	0.00**	0.00	[0,0]	0.00**	0.00	[0,0]
Professional/administration occupation	—	—	—	0.00	0.00	[-0.01,0.01]	0.00	0.00	[-0.01,0.01]	0.00	0.00	[-0.01,0.01]
ADL-related disability	—	—	—	-0.01***	0.00	[-0.01,0]	-0.01**	0.00	[-0.01,0]	-0.01**	0.00	[-0.01,0]
Subjective health	—	—	—	0.01***	0.00	[0,0.01]	0.01**	0.00	[0,0.01]	0.01**	0.00	[0,0.01]
C2	0.03***	0.01	[0.02,0.04]	—	—	—	0.02*	0.01	[0,0.03]	0.00	0.01	[-0.02,0.02]
C3	0.04***	0.01	[0.03,0.06]	—	—	—	0.02**	0.01	[0.01,0.04]	0.02	0.01	[-0.01,0.04]
C4	0.04***	0.01	[0.02,0.05]	—	—	—	0.02**	0.01	[0.01,0.04]	0.01	0.01	[-0.01,0.03]
C5	0.02**	0.01	[0.01,0.04]	—	—	—	0.01	0.01	[-0.01,0.03]	-0.01	0.02	[-0.04,0.02]
C2×Male	—	—	—	—	—	—	—	—	—	0.02†	0.01	[0,0.05]
C3×Male	—	—	—	—	—	—	—	—	—	0.02	0.01	[-0.01,0.04]
C4×Male	—	—	—	—	—	—	—	—	—	0.02	0.01	[-0.01,0.05]
C5×Male	—	—	—	—	—	—	—	—	—	0.03†	0.02	[0,0.07]

Note. †$p < .10$, *$p < .05$, **$p < .01$, ***$p < .001$.

Supplementary Table S7. Regression models of general cognition among the old-old group.

Variable	M0			M1			M2			M3		
	β	*SE*	95% CI	β	*SE*	95% CI	β	*SE*	95% CI	β	*SE*	95% CI
Age	—	—	—	-0.10***	0.01	[-0.11,-0.08]	-0.08***	0.01	[-0.09,-0.06]	-0.08***	0.01	[-0.09,-0.06]
Male	—	—	—	0.49***	0.08	[0.33,0.65]	0.44***	0.08	[0.28,0.6]	0.54**	0.16	[0.22,0.86]
Living alone	—	—	—	-0.06	0.10	[-0.25,0.13]	-0.07	0.10	[-0.26,0.11]	-0.07	0.10	[-0.26,0.11]
Urban residence	—	—	—	0.06	0.10	[-0.14,0.25]	-0.13	0.10	[-0.33,0.07]	-0.14	0.10	[-0.33,0.06]
Years of education	—	—	—	0.12***	0.02	[0.09,0.15]	0.10***	0.02	[0.07,0.13]	0.10***	0.02	[0.07,0.13]
Professional/administration occupation	—	—	—	0.46**	0.17	[0.13,0.79]	0.34*	0.17	[0.01,0.67]	0.35*	0.17	[0.03,0.68]
ADL-related disability	—	—	—	-0.20***	0.03	[-0.26,-0.15]	-0.12***	0.03	[-0.18,-0.07]	-0.12***	0.03	[-0.18,-0.07]
Subjective health	—	—	—	0.35***	0.04	[0.27,0.43]	0.29***	0.04	[0.21,0.37]	0.29***	0.04	[0.21,0.37]
C2	1.26***	0.10	[1.06,1.46]	—	—	—	0.85***	0.11	[0.63,1.06]	0.85***	0.14	[0.58,1.11]
C3	2.67***	0.14	[2.39,2.95]	—	—	—	1.68***	0.15	[1.38,1.98]	2.09***	0.24	[1.62,2.55]
C4	1.65***	0.13	[1.41,1.9]	—	—	—	1.06***	0.13	[0.81,1.32]	1.09***	0.18	[0.73,1.45]
C5	1.11***	0.13	[0.85,1.37]	—	—	—	0.65***	0.13	[0.39,0.91]	0.76***	0.20	[0.38,1.14]
C2×Male	—	—	—	—	—	—	—	—	—	-0.01	0.20	[-0.41,0.4]
C3×Male	—	—	—	—	—	—	—	—	—	-0.64*	0.30	[-1.22,-0.07]
C4×Male	—	—	—	—	—	—	—	—	—	-0.07	0.25	[-0.57,0.42]
C5×Male	—	—	—	—	—	—	—	—	—	-0.22	0.27	[-0.74,0.3]

Note. $^{+}p < .10$, $^{*}p < .05$, $^{**}p < .01$, $^{***}p < .001$.

Supplementary Table S8. Regression models of orientation ability among the old-old group.

Variable	M0			M1			M2			M3		
	β	*SE*	95% CI	β	*SE*	95% CI	β	*SE*	95% CI	β	*SE*	95% CI
Age	—	—	—	0.00^{***}	0.00	[0,0]	0.00^{***}	0.00	[0,0]	0.00^{***}	0.00	[0,0]
Male	—	—	—	0.01^{***}	0.00	[0.01,0.02]	0.01^{***}	0.00	[0.01,0.02]	0.01	0.01	[0,0.02]
Living alone	—	—	—	0.01^{*}	0.00	[0,0.01]	0.01^{*}	0.00	[0,0.02]	0.01^{*}	0.00	[0,0.02]
Urban residence	—	—	—	-0.01^{**}	0.00	[-0.02,0]	-0.01^{***}	0.00	[-0.02,-0.01]	-0.01^{***}	0.00	[-0.02,-0.01]
Years of education	—	—	—	0.00^{***}	0.00	[0,0]	0.00^{***}	0.00	[0,0]	0.00^{***}	0.00	[0,0]
Professional/administration occupation	—	—	—	0.00	0.01	[-0.01,0.01]	0.00	0.01	[-0.01,0.01]	0.00	0.01	[-0.01,0.01]
ADL-related disability	—	—	—	-0.01^{***}	0.00	[-0.01,-0.01]	-0.01^{***}	0.00	[-0.01,-0.01]	-0.01^{***}	0.00	[-0.01,-0.01]
Subjective health	—	—	—	0.01^{***}	0.00	[0.01,0.01]	0.01^{***}	0.00	[0.01,0.01]	0.01^{***}	0.00	[0.01,0.01]
C2	0.02^{***}	0.00	[0.02,0.03]	—	—	—	$0.01^{\dagger}$	0.00	[0,0.01]	0.01	0.01	[-0.01,0.02]
C3	0.05^{***}	0.01	[0.04,0.06]	—	—	—	0.02^{**}	0.01	[0.01,0.03]	0.02^{*}	0.01	[0,0.04]
C4	0.03^{***}	0.01	[0.03,0.04]	—	—	—	0.02^{***}	0.01	[0.01,0.03]	0.01^{*}	0.01	[0,0.03]
C5	0.02^{***}	0.00	[0.02,0.03]	—	—	—	0.01^{*}	0.01	[0,0.02]	$0.01^{\dagger}$	0.01	[0,0.03]
C2×Male	—	—	—	—	—	—	—	—	—	0.00	0.01	[-0.01,0.02]
C3×Male	—	—	—	—	—	—	—	—	—	0.00	0.01	[-0.02,0.02]
C4×Male	—	—	—	—	—	—	—	—	—	0.01	0.01	[-0.01,0.02]
C5×Male	—	—	—	—	—	—	—	—	—	0.00	0.01	[-0.02,0.02]

Note. $^{\dagger}p < .10$, $^{*}p < .05$, $^{**}p < .01$, $^{***}p < .001$.

Supplementary Table S9. Regression models of calculation ability among the old-old group.

Variable	M0			M1			M2			M3		
	β	*SE*	95% CI	β	*SE*	95% CI	β	*SE*	95% CI	β	*SE*	95% CI
Age	—	—	—	-0.01***	0.00	[-0.01,0]	0.00***	0.00	[-0.01,0]	0.00***	0.00	[-0.01,0]
Male	—	—	—	0.10***	0.01	[0.09,0.12]	0.10***	0.01	[0.08,0.11]	0.11***	0.02	[0.08,0.14]
Living alone	—	—	—	0.02†	0.01	[0,0.03]	0.02†	0.01	[0,0.03]	0.02†	0.01	[0,0.04]
Urban residence	—	—	—	0.02	0.01	[0,0.03]	0.01	0.01	[-0.01,0.03]	0.01	0.01	[-0.01,0.02]
Years of education	—	—	—	0.01***	0.00	[0.01,0.01]	0.01***	0.00	[0,0.01]	0.01***	0.00	[0,0.01]
Professional/administration occupation	—	—	—	0.01	0.02	[-0.02,0.04]	0.00	0.02	[-0.03,0.03]	0.00	0.02	[-0.03,0.04]
ADL-related disability	—	—	—	-0.01***	0.00	[-0.02,-0.01]	-0.01**	0.00	[-0.01,0]	-0.01**	0.00	[-0.01,0]
Subjective health	—	—	—	0.02***	0.00	[0.01,0.03]	0.02***	0.00	[0.01,0.02]	0.02***	0.00	[0.01,0.02]
C2	0.07***	0.01	[0.05,0.09]	—	—	—	0.04***	0.01	[0.02,0.07]	0.05**	0.01	[0.02,0.07]
C3	0.17***	0.01	[0.14,0.19]	—	—	—	0.09***	0.01	[0.06,0.11]	0.13***	0.02	[0.08,0.17]
C4	0.10***	0.01	[0.07,0.12]	—	—	—	0.05***	0.01	[0.02,0.07]	0.05**	0.02	[0.02,0.09]
C5	0.10***	0.01	[0.08,0.13]	—	—	—	0.06***	0.01	[0.04,0.09]	0.08***	0.02	[0.04,0.12]
C2×Male	—	—	—	—	—	—	—	—	—	0.00	0.02	[-0.04,0.04]
C3×Male	—	—	—	—	—	—	—	—	—	-0.06*	0.03	[-0.12,-0.01]
C4×Male	—	—	—	—	—	—	—	—	—	-0.01	0.02	[-0.05,0.04]
C5×Male	—	—	—	—	—	—	—	—	—	-0.04	0.03	[-0.09,0.01]

Note. †$p < .10$, *$p < .05$, **$p < .01$, ***$p < .001$.

Supplementary Table S10. Regression models of recall ability among the old-old group.

Variable	M0			M1			M2			M3		
	β	*SE*	95% CI	β	*SE*	95% CI	β	*SE*	95% CI	β	*SE*	95% CI
Age	—	—	—	0.00***	0.00	[-0.01,0]	0.00**	0.00	[0,0]	0.00**	0.00	[0,0]
Male	—	—	—	0.03**	0.01	[0.01,0.05]	0.03**	0.01	[0.01,0.05]	0.04†	0.02	[0,0.08]
Living alone	—	—	—	0.01	0.01	[-0.01,0.04]	0.01	0.01	[-0.01,0.04]	0.01	0.01	[-0.01,0.04]
Urban residence	—	—	—	-0.01	0.01	[-0.04,0.01]	-0.02†	0.01	[-0.04,0]	-0.02†	0.01	[-0.05,0]
Years of education	—	—	—	0.01***	0.00	[0.01,0.01]	0.01***	0.00	[0,0.01]	0.01***	0.00	[0,0.01]
Professional/administration occupation	—	—	—	0.04*	0.02	[0,0.08]	0.03†	0.02	[-0.01,0.07]	0.03†	0.02	[-0.01,0.07]
ADL-related disability	—	—	—	-0.01**	0.00	[-0.02,0]	-0.01*	0.00	[-0.01,0]	-0.01*	0.00	[-0.01,0]
Subjective health	—	—	—	0.03***	0.01	[0.02,0.04]	0.03***	0.01	[0.02,0.04]	0.03***	0.01	[0.02,0.04]
C2	0.05***	0.01	[0.03,0.08]	—	—	—	0.03*	0.01	[0,0.05]	0.03*	0.02	[0,0.07]
C3	0.16***	0.02	[0.13,0.19]	—	—	—	0.10***	0.02	[0.06,0.13]	0.12***	0.03	[0.07,0.17]
C4	0.06***	0.02	[0.03,0.09]	—	—	—	0.02	0.02	[-0.01,0.05]	0.03	0.02	[-0.01,0.07]
C5	0.05**	0.02	[0.02,0.08]	—	—	—	0.02	0.02	[-0.02,0.05]	0.01	0.02	[-0.04,0.06]
C2×Male	—	—	—	—	—	—	—	—	—	-0.02	0.03	[-0.07,0.03]
C3×Male	—	—	—	—	—	—	—	—	—	-0.04	0.04	[-0.11,0.03]
C4×Male	—	—	—	—	—	—	—	—	—	-0.02	0.03	[-0.08,0.04]
C5×Male	—	—	—	—	—	—	—	—	—	0.01	0.03	[-0.05,0.07]

Note. †*p*< .10, **p*< .05, ***p*< .01, ****p*< .001.

Supplementary Table S11. Regression models of language ability among the old-old group.

Variable	M0			M1			M2			M3		
	β	*SE*	95% CI	β	*SE*	95% CI	β	*SE*	95% CI	β	*SE*	95% CI
Age	—	—	—	0.00***	0.00	[0,0]	0.00***	0.00	[0,0]	0.00***	0.00	[0,0]
Male	—	—	—	0.01*	0.00	[0,0.02]	0.01	0.00	[0,0.01]	0.00	0.01	[-0.02,0.01]
Living alone	—	—	—	0.01**	0.00	[0.01,0.02]	0.01**	0.00	[0.01,0.02]	0.01**	0.00	[0.01,0.02]
Urban residence	—	—	—	0.01**	0.01	[0.01,0.02]	0.01†	0.01	[0,0.02]	0.01†	0.01	[0,0.02]
Years of education	—	—	—	0.00***	0.00	[0,0]	0.00***	0.00	[0,0]	0.00***	0.00	[0,0]
Professional/administration occupation	—	—	—	0.02†	0.01	[0,0.03]	0.01	0.01	[0,0.03]	0.01†	0.01	[0,0.03]
ADL-related disability	—	—	—	-0.01***	0.00	[-0.01,0]	-0.01***	0.00	[-0.01,0]	-0.01***	0.00	[-0.01,0]
Subjective health	—	—	—	0.01***	0.00	[0.01,0.02]	0.01***	0.00	[0.01,0.02]	0.01***	0.00	[0.01,0.02]
C2	0.01**	0.00	[0.01,0.02]	—	—	—	0.00	0.01	[-0.01,0.01]	-0.01	0.01	[-0.02,0.01]
C3	0.05***	0.01	[0.04,0.06]	—	—	—	0.02*	0.01	[0,0.03]	0.03*	0.01	[0.01,0.05]
C4	0.05***	0.01	[0.04,0.06]	—	—	—	0.03***	0.01	[0.01,0.04]	0.02*	0.01	[0,0.04]
C5	0.02**	0.01	[0.01,0.03]	—	—	—	0.01	0.01	[-0.01,0.02]	0.00	0.01	[-0.02,0.02]
C2×Male	—	—	—	—	—	—	—	—	—	0.01	0.01	[-0.01,0.03]
C3×Male	—	—	—	—	—	—	—	—	—	-0.01	0.01	[-0.04,0.02]
C4×Male	—	—	—	—	—	—	—	—	—	0.01	0.01	[-0.01,0.03]
C5×Male	—	—	—	—	—	—	—	—	—	0.01	0.01	[-0.02,0.03]

Note. †p< .10, *p< .05, **p< .01, ***p< .001.

Supplementary Table S12. Regression models of general cognition among the oldest-old group.

Variable	M0			M1			M2			M3		
	β	*SE*	95% CI	β	*SE*	95% CI	β	*SE*	95% CI	β	*SE*	95% CI
Age	—	—	—	-0.06	0.04	[-0.15,0.02]	-0.06	0.04	[-0.14,0.03]	-0.06	0.04	[-0.14,0.03]
Male	—	—	—	0.80**	0.25	[0.32,1.28]	0.80**	0.25	[0.31,1.28]	1.06**	0.38	[0.31,1.81]
Living alone	—	—	—	0.08	0.31	[-0.54,0.69]	-0.13	0.31	[-0.74,0.48]	-0.12	0.31	[-0.73,0.49]
Urban residence	—	—	—	0.42†	0.23	[-0.03,0.88]	0.35	0.23	[-0.11,0.81]	0.37	0.24	[-0.09,0.83]
Years of education	—	—	—	-0.02	0.05	[-0.12,0.08]	-0.03	0.05	[-0.12,0.07]	-0.03	0.05	[-0.12,0.07]
Professional/administration occupation	—	—	—	1.18†	0.61	[-0.01,2.37]	1.04†	0.60	[-0.13,2.21]	1.11†	0.60	[-0.07,2.28]
ADL-related disability	—	—	—	-0.17***	0.04	[-0.26,-0.09]	-0.07	0.05	[-0.16,0.02]	-0.07	0.05	[-0.16,0.02]
Subjective health	—	—	—	0.53***	0.12	[0.30,0.75]	0.48***	0.12	[0.26,0.71]	0.48***	0.12	[0.25,0.70]
C2	1.52***	0.24	[1.06,1.98]	—	—	—	1.50***	0.25	[1.01,1.99]	1.51***	0.27	[0.98,2.04]
C3	3.06***	0.50	[2.07,4.05]	—	—	—	2.35***	0.52	[1.33,3.37]	2.41**	0.70	[1.04,3.79]
C4	1.31***	0.30	[0.73,1.90]	—	—	—	0.83**	0.31	[0.22,1.44]	1.03**	0.38	[0.28,1.78]
C5	0.77**	0.28	[0.22,1.32]	—	—	—	0.45	0.29	[-0.12,1.02]	0.66†	0.35	[-0.03,1.34]
C2×Male	—	—	—	—	—	—	—	—	—	0.04	0.65	[-1.23,1.30]
C3×Male	—	—	—	—	—	—	—	—	—	-0.31	1.02	[-2.32,1.70]
C4×Male	—	—	—	—	—	—	—	—	—	-0.62	0.64	[-1.88,0.64]
C5×Male	—	—	—	—	—	—	—	—	—	-0.71	0.63	[-1.94,0.52]

Note. $^{\dagger}p < .10$, $^{*}p < .05$, $^{**}p < .01$, $^{***}p < .001$. The regression coefficients in Tables 1–10 are unstandardized betas.

Commented [KW1]: Please confirm consistency with the footer of Table 2.

Supplementary Table S13. Regression models of orientation ability among the oldest-old group.

Variable	M0			M1			M2			M3		
	β	*SE*	95% CI	β	*SE*	95% CI	β	*SE*	95% CI	β	*SE*	95% CI
Age	—	—	—	0.00	0.00	[-0.01,0]	0.00	0.00	[-0.01,0]	0.00	0.00	[-0.01,0]
Male	—	—	—	0.02*	0.01	[0,0.04]	0.02*	0.01	[0,0.04]	0.02	0.02	[-0.01,0.05]
Living alone	—	—	—	0.01	0.01	[-0.01,0.04]	0.01	0.01	[-0.01,0.04]	0.01	0.01	[-0.01,0.04]
Urban residence	—	—	—	-0.02**	0.01	[-0.04,-0.01]	-0.03**	0.01	[-0.04,-0.01]	-0.03**	0.01	[-0.04,-0.01]
Years of education	—	—	—	0.00	0.00	[0,0]	0.00	0.00	[0,0]	0.00	0.00	[0,0]
Professional/administration occupation	—	—	—	0.02	0.02	[-0.02,0.07]	0.02	0.02	[-0.03,0.07]	0.02	0.02	[-0.03,0.07]
ADL-related disability	—	—	—	-0.01***	0.00	[-0.01,0]	-0.01**	0.00	[-0.01,0]	-0.01**	0.00	[-0.01,0]
Subjective health	—	—	—	0.02***	0.00	[0.01,0.02]	0.02**	0.00	[0.01,0.02]	0.02**	0.01	[0.01,0.02]
C2	0.03**	0.01	[0.01,0.05]	—	—	—	0.01	0.01	[0,0.02]	0.02	0.01	[-0.01,0.04]
C3	0.05*	0.02	[0.01,0.09]	—	—	—	0.03	0.02	[-0.01,0.07]	0.04	0.03	[-0.02,0.09]
C4	0.02†	0.01	[0,0.04]	—	—	—	0.00	0.01	[-0.02,0.03]	0.00	0.02	[-0.03,0.03]
C5	0.01	0.01	[-0.01,0.03]	—	—	—	0.01	0.01	[-0.02,0.03]	0.00	0.01	[-0.03,0.03]
C2×Male	—	—	—	—	—	—	—	—	—	-0.01	0.03	[-0.06,0.04]
C3×Male	—	—	—	—	—	—	—	—	—	-0.01	0.04	[-0.09,0.07]
C4×Male	—	—	—	—	—	—	—	—	—	0.00	0.03	[-0.05,0.05]
C5×Male	—	—	—	—	—	—	—	—	—	0.01	0.03	[-0.03,0.06]

Note. †$p < .10$, *$p < .05$, **$p < .01$, ***$p < .001$.

Supplementary Table S14. Regression models of calculation ability among the oldest-old group.

Variable	M0			M1			M2			M3		
	β	*SE*	95% CI	β	*SE*	95% CI	β	*SE*	95% CI	β	*SE*	95% CI
Age	—	—	—	0.00	0.00	[-0.01,0.01]	0.00	0.00	[-0.01,0.01]	0.00	0.00	[-0.01,0.01]
Male	—	—	—	0.10***	0.03	[0.05,0.15]	0.10***	0.03	[0.05,0.15]	0.08†	0.04	[0,0.15]
Living alone	—	—	—	0.04	0.03	[-0.02,0.10]	0.03	0.03	[-0.03,0.10]	0.04	0.03	[-0.03,0.10]
Urban residence	—	—	—	0.00	0.02	[-0.05,0.04]	0.00	0.02	[-0.05,0.05]	0.00	0.02	[-0.05,0.05]
Years of education	—	—	—	0.00	0.01	[-0.01,0.01]	0.00	0.01	[-0.01,0.01]	0.00	0.01	[-0.01,0.01]
Professional/administration occupation	—	—	—	0.10†	0.06	[-0.01,0.22]	0.10†	0.06	[-0.01,0.22]	0.10†	0.06	[-0.01,0.22]
ADL-related disability	—	—	—	-0.02***	0.01	[-0.03,-0.01]	-0.02**	0.01	[-0.03,-0.01]	-0.02**	0.01	[-0.03,-0.01]
Subjective health	—	—	—	0.02	0.01	[-0.01,0.04]	0.01	0.01	[-0.01,0.04]	0.01	0.01	[-0.01,0.04]
C2	0.07**	0.02	[0.03,0.12]	—	—	—	0.05*	0.03	[0,0.10]	0.04	0.03	[-0.02,0.10]
C3	0.09†	0.05	[0,0.19]	—	—	—	0.02	0.05	[-0.08,0.12]	-0.02	0.07	[-0.16,0.11]
C4	0.11**	0.03	[0.05,0.17]	—	—	—	0.05	0.03	[-0.01,0.12]	0.06	0.04	[-0.02,0.14]
C5	0.04	0.03	[-0.02,0.10]	—	—	—	0.00	0.03	[-0.06,0.06]	0.00	0.04	[-0.07,0.07]
C2×Male	—	—	—	—	—	—	—	—	—	0.08	0.07	[-0.05,0.21]
C3×Male	—	—	—	—	—	—	—	—	—	0.10	0.10	[-0.10,0.30]
C4×Male	—	—	—	—	—	—	—	—	—	0.01	0.07	[-0.13,0.14]
C5×Male	—	—	—	—	—	—	—	—	—	0.01	0.07	[-0.12,0.14]

Note. †p< .10, *p< .05, **p< .01, ***p< .001.

Supplementary Table S15. Regression models of recall ability among the oldest-old group.

Variable	M0			M1			M2			M3		
	β	*SE*	95% CI	β	*SE*	95% CI	β	*SE*	95% CI	β	*SE*	95% CI
Age	—	—	—	-0.02**	0.01	[-0.03,-0.01]	-0.02**	0.01	[-0.03,-0.01]	-0.02**	0.01	[-0.03,-0.01]
Male	—	—	—	0.01	0.03	[-0.05,0.06]	0.01	0.03	[-0.05,0.06]	0.02	0.05	[-0.07,0.10]
Living alone	—	—	—	0.03	0.04	[-0.04,0.10]	0.03	0.04	[-0.04,0.10]	0.03	0.04	[-0.04,0.10]
Urban residence	—	—	—	0.01	0.03	[-0.04,0.06]	0.01	0.03	[-0.05,0.06]	0.00	0.03	[-0.05,0.06]
Years of education	—	—	—	-0.01	0.01	[-0.02,0]	-0.01	0.01	[-0.02,0]	-0.01	0.01	[-0.02,0]
Professional/administration occupation	—	—	—	0.13†	0.07	[0,0.25]	0.12†	0.07	[-0.01,0.25]	0.12†	0.07	[-0.01,0.25]
ADL-related disability	—	—	—	-0.02***	0.01	[-0.03,-0.01]	-0.02***	0.01	[-0.03,-0.01]	-0.02***	0.01	[-0.03,-0.01]
Subjective health	—	—	—	0.03*	0.01	[0,0.06]	0.03*	0.01	[0,0.06]	0.03*	0.01	[0,0.06]
C2	0.09**	0.03	[0.04,0.14]	—	—	—	0.05	0.03	[-0.01,0.10]	0.05†	0.03	[-0.01,0.11]
C3	0.15**	0.05	[0.04,0.25]	—	—	—	0.09	0.06	[-0.02,0.20]	0.11	0.08	[-0.04,0.26]
C4	0.06†	0.03	[0,0.13]	—	—	—	0.01	0.04	[-0.06,0.08]	0.00	0.04	[-0.09,0.08]
C5	0.06†	0.03	[-0.01,0.12]	—	—	—	0.04	0.03	[-0.03,0.10]	0.05	0.04	[-0.03,0.12]
C2×Male	—	—	—	—	—	—	—	—	—	-0.03	0.07	[-0.17,0.11]
C3×Male	—	—	—	—	—	—	—	—	—	-0.05	0.11	[-0.26,0.17]
C4×Male	—	—	—	—	—	—	—	—	—	0.03	0.07	[-0.11,0.18]
C5×Male	—	—	—	—	—	—	—	—	—	-0.03	0.07	[-0.17,0.11]

Note. †p< .10, *p< .05, **p< .01, ***p< .001.

Supplementary Table S16. Regression models of language ability among the oldest-old group.

Variable	M0			M1			M2			M3		
	β	*SE*	95% CI	β	*SE*	95% CI	*β*	*SE*	95% CI	β	*SE*	95% CI
Age	—	—	—	$0.00^{†}$	0.00	[-0.01,0]	$0.00^{†}$	0.00	[-0.01,0]	$0.00^{†}$	0.00	[-0.01,0]
Male	—	—	—	0.02	0.01	[-0.01,0.04]	0.02	0.01	[-0.01,0.04]	0.01	0.02	[-0.03,0.05]
Living alone	—	—	—	0.04^{**}	0.02	[0.01,0.07]	0.04^{**}	0.02	[0.01,0.07]	0.04^{**}	0.02	[0.01,0.07]
Urban residence	—	—	—	0.01	0.01	[-0.01,0.03]	0.01	0.01	[-0.01,0.03]	0.01	0.01	[-0.01,0.03]
Years of education	—	—	—	0.00	0.00	[0,0.01]	0.00	0.00	[0,0.01]	0.00	0.00	[0,0.01]
Professional/administration occupation	—	—	—	0.03	0.03	[-0.02,0.09]	0.04	0.03	[-0.02,0.09]	0.04	0.03	[-0.02,0.10]
ADL-related disability	—	—	—	-0.01^{**}	0.00	[-0.01,0]	-0.01^{**}	0.00	[-0.01,0]	-0.01^{**}	0.00	[-0.01,0]
Subjective health	—	—	—	0.02^{**}	0.01	[0,0.03]	0.02^{**}	0.01	[0.01,0.03]	0.02^{**}	0.01	[0,0.03]
C2	0.02	0.01	[-0.01,0.04]	—	—	—	0.00	0.01	[-0.03,0.02]	0.00	0.01	[-0.03,0.02]
C3	-0.02	0.02	[-0.07,0.03]	—	—	—	-0.06^{*}	0.03	[-0.11,-0.01]	-0.10^{**}	0.03	[-0.17,-0.04]
C4	$0.03^{†}$	0.01	[0,0.05]	—	—	—	0.00	0.02	[-0.03,0.03]	0.00	0.02	[-0.04,0.04]
C5	0.01	0.01	[-0.02,0.03]	—	—	—	-0.01	0.01	[-0.04,0.02]	0.00	0.02	[-0.03,0.03]
C2×Male	—	—	—	—	—	—	—	—	—	0.02	0.03	[-0.05,0.08]
C3×Male	—	—	—	—	—	—	—	—	—	$0.10^{†}$	0.05	[0,0.19]
C4×Male	—	—	—	—	—	—	—	—	—	0.00	0.03	[-0.06,0.06]
C5×Male	—	—	—	—	—	—	—	—	—	-0.02	0.03	[-0.08,0.04]

Note. $^{†}p< .10$, $^{*}p< .05$, $^{**}p< .01$, $^{***}p< .001$.

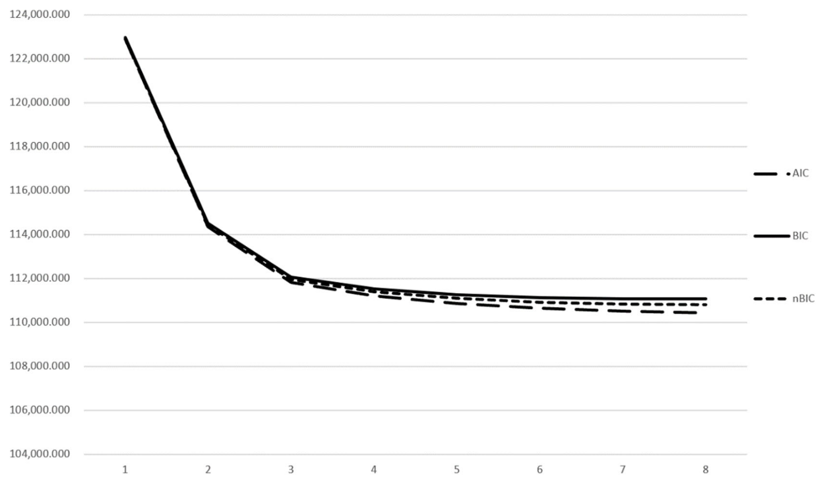

Supplementary Figure S1. Information criteria for 1–8 class model.
